# Genomics of Hospital-Associated Brazilian Multidrug-Resistant *Klebsiella pneumoniae*: Abundance of Resistance and Virulence Genes and Mosaicism of the *bla*_KPC−2_ Genetic Context Among *Enterobacterales*

**DOI:** 10.1007/s00284-026-04989-w

**Published:** 2026-06-10

**Authors:** Monalessa Fábia Pereira, Ciro César Rossi, Mirla Borghi, Beatriz Dias Januário, Ana Luisa Andrade-Oliveira, Denise Mara Soares Bazzolli, Luiz Gonzaga Paula de Almeida, Ana Tereza Ribeiro de Vasconcelos, Marisa Fabiana Nicolás, Ricardo Pinto Schuenck

**Affiliations:** 1 Instituto de Biodiversidade e Sustentabilidade, Universidade Federal do Rio de Janeiro, Macaé, RJ Brazil; 2 Departamento de Ciências Biológicas, Universidade do Estado de Minas Gerais, Carangola, MG Brazil; 3 Departamento de Biologia Celular e Molecular, Universidade Federal Fluminense, Niterói, RJ Brazil; 4 Departamento de Patologia, Universidade Federal do Espírito Santo, Vitória, ES Brazil; 5 Instituto de Microbiologia Paulo de Góes, Universidade Federal do Rio de Janeiro, Rio de Janeiro, RJ Brazil; 6 Departamento de Microbiologia, Universidade Federal de Viçosa, Viçosa, MG Brazil; 7 Laboratório Nacional de Computação Científica, Petrópolis, RJ Brazil

## Abstract

The emergence of carbapenem-resistant *Klebsiella pneumoniae* (CRKP) poses a critical threat to global public health due to limited therapeutic options. This situation is magnified by CRKP strains with elevated virulence. This study aimed to characterize the virulome, resistome, and *bla*_KPC_ genetic context of CRKP strains exhibiting increased virulence from hospital-associated infections in southeastern Brazil, focusing on their molecular evolution and clinical impact. Despite being classified as classical variants, the strains displayed a dense virulome, averaging 14 ± 0.55 virulence genes, many linked to mobile genetic elements and co-occurring with heavy metal resistance genes. Notably, the colicin-encoding *cci* gene, reported for the first time in ST147, illustrates unique adaptations in this lineage. Diversity was observed in K- and O-loci, including the rare K-locus 150, identified in an ST11 strain featuring a rearrangement involving the virulence-associated fucose synthesis gene *gmb*. The pan-resistome included 51 acquired resistance genes (ARGs), with an average of 14.7 ± 2.8 per strain, enabling resistance to multiple antibiotic classes. The colocalization of ARGs suggests horizontal gene transfer as a driver of resistance dissemination. All *bla*_KPC−2_-carrying strains also contained ESBL genes, with the *bla*_KPC−2_ gene typically located on IncN or IncM1-type plasmids within Tn*4401*, a conserved genetic context. However, an unusual *bla*_KPC−2_ context, associated with Tn*5403* and suggesting a putative recombination event between plasmids from different Proteobacteria, was found in an ST11 (CC258) strain. These findings highlight the urgent need for genomic surveillance in hospitals to monitor and understand the evolution of resistance and virulence in CRKP.

**Supplementary Information:**

The online version contains supplementary material available at 10.1007/s00284-026-04989-w.

## Introduction

*Klebsiella pneumoniae* is a major opportunistic pathogen traditionally associated with a broad spectrum of infections, including pneumonia, urinary tract, wound, and bloodstream infections, particularly in immunocompromised patients and individuals frequently exposed to healthcare settings [1].

The increasing prevalence of multidrug-resistant (MDR) *K. pneumoniae* strains has drastically limited therapeutic options and is associated with heightened morbidity and mortality rates [2]. Over the past decade, resistance to third-generation cephalosporins has increased reliance on carbapenems as last-line agents against MDR *K. pneumoniae*, driving the emergence of carbapenem-resistant strains [3]. This resistance is primarily mediated by plasmids and transposons encoding carbapenemase genes, such *bla*_KPC_, which facilitates the rapid dissemination of this genetic information across bacterial populations [4]. For this reason, carbapenem-resistant *K. pneumoniae* is classified by the World Health Organization (WHO) as a critical public health threat requiring novel control approaches [5].

In the context of virulence, *K. pneumoniae* strains are classified as classical (cKp) and hypervirulent (hvKp), with most *K. pneumoniae* infections attributed to cKp strains [6]. Among the most important virulence factors of *K. pneumoniae* are the polysaccharide capsule, O antigen, adhesive pili, regulators of capsule overproduction, and siderophores such as enterobactin, aerobactin, salmochelin, and yersiniabactin, which are encoded in both the core and accessory genomes, the latter often acquired via horizontal gene transfer [2]. Among these, the genes responsible for capsule overproduction and the siderophores aerobactin and salmochelin serve as markers of hypervirulence, and are typically absent in most cKp strains [7].

Although certain aspects of the virulence of carbapenem-resistant strains remain poorly understood, the combination of antimicrobial resistance and virulence factors clearly represents a serious threat to public health. In this context, this study aimed to characterize the resistome, virulome, and genetic context of *bla*_KPC_ in CRKP strains previously isolated from hospital-associated infections in southeastern Brazil, with the goal of understanding the molecular evolution of these lineages and highlighting the clinical significance of these findings.

## Materials and Methods

### Bacterial Strains

This study was conducted with 11 carbapenem-resistant *Klebsiella pneumoniae* (CRKP) strains selected from a previously characterized collection of 40 clinical isolates, as described in Borghi et al. [8]. These isolates were obtained from patients admitted to hospitals in the metropolitan region of Vitória (20°19′8″S, 40°20′16″W), Espírito Santo, southeastern Brazil, between April and November 2015. All isolates were derived from different patients and diverse clinical sources and were classified as multidrug-resistant, exhibiting resistance to at least one agent in three or more antimicrobial classes, including carbapenems. Antimicrobial susceptibility testing (AST) data for these isolates were previously determined and reported in Borghi et al. [8], following EUCAST guidelines.

The strains included in this study were selected to represent distinct sequence types and pulsotypes identified in the original dataset, including high-risk clones such as those belonging to clonal complex 258 (CC258). These strains were selected for genomic characterization due to the prior observation of their diverse sequence types and alarming phenotypes of resistance to last-resort antimicrobials and virulence in the *Galleria mellonella* infection model [8].

### Extraction, Sequencing and Annotation of Genomic DNA

The 11 CRKP strains were grown separately on BHI agar (BD Biosciences, USA) for 24 h. Total genomic DNA of each strain was extracted using the FastDNA Spin kit (MP Biomedicals, USA), according to the manufacturer’s instructions. Genomic DNA paired-end libraries were then generated using the TruSeq DNA Nano sample preparation kit (Illumina Inc, EUA) and sequenced on the Illumina HiSeq 2500 platform, producing 2 × 150-bp paired-end reads (Illumina Inc, EUA). The quality of the high-throughput sequence data was assessed by FastQC [9]. The short reads were assembled *de novo* into contigs using SPAdes 3.9.0 [10], and the assembly quality was evaluated by QUAST [11]. Scaffolding was performed with Newbler v 2.6 (Roche Inc.) [10], and sequence gaps were closed with GapFiller [12].

The initial gene prediction was carried out using GeneMark [13]. Subsequently, automatic annotation of predicted genes was performed using BLASTp against the NCBI-nr (www.ncbi.nlm.nih.gov/protein), KEGG (www.genome.jp/kegg), UniProt (www.uniprot.org), and TCDB (www.tcdb.org) databases using 100% query and subject coverage and a minimum of 95% identity as the cutoff.

### Genomic Analysis of Virulence and Antimicrobial Resistance

Capsule synthesis loci (K-loci) and lipopolysaccharide synthesis loci (O-loci) were characterized using the Kaptive 2.0 tool [14]. Virulence genes were identified with the BIGSdb-Kp (Institut Pasteur) [15] and VirulenceFinder 2.0 databases [16]. Genes associated with heavy metal resistance were also identified using the BIGSdb-Kp.

Acquired resistance genes (ARGs) and chromosomal mutations mediating antimicrobial resistance were analyzed with ResFinder [17], using an identity threshold of 98%. Efflux systems and their regulators were identified through BIGSdb-Kp.

The MobileElementFinder tool [18] was used to identify mobile genetic elements and their association with antimicrobial resistance genes and virulence factors. To extract plasmid sequences from the genome assemblies, the MOB-recon tool available in MOB Suite v3.0.3 was used [19]. Plasmid assemblies were also used as inputs to StarAMR [20] to identify plasmids carrying the *bla*_KPC−2_ gene.

### Phylogenetic Analysis

Phylogenetic relationships among the CRKP isolates were inferred using the concatenated alignment of seven housekeeping genes (*gapA*, *infB*, *mdh*, *pgi*, *phoE*, *rpoB*, and *tonB*) used in multilocus sequence typing (MLST). Sequence alignments were performed, and phylogenetic trees were reconstructed using Bayesian inference implemented in MrBayes v3.2.7a with default parameters. The resulting tree was visualized and used to map the distribution of virulence and resistance determinants across the analyzed isolates.

## Results

### Nucleotide Sequence Accession Numbers and Genomic Features of CRKP Strains

The BioProject accession number for the genomes of the 11 CRKP strains in GenBank/NCBI is PRJNA1240238. The sequenced CRKP genomes exhibited high coverage and an average size of 5.32 ± 0.3 Mbp, with a GC content of 57.5% ± 0.31% and coding regions averaging 844.97 ± 9.26 bp. Each genome contained approximately 5570 ± 42.01 protein-coding genes. Genomic analysis revealed that strain 19B belongs to the species *Klebsiella quasipneumoniae*, while the other strains, consistent with previous tests, were identified as *K. pneumoniae*. Both species belong to the *K. pneumoniae* complex.

### Diversity and Genetic Features of K-Loci and O-Loci in CRKP Strains

**Fig. 1:**
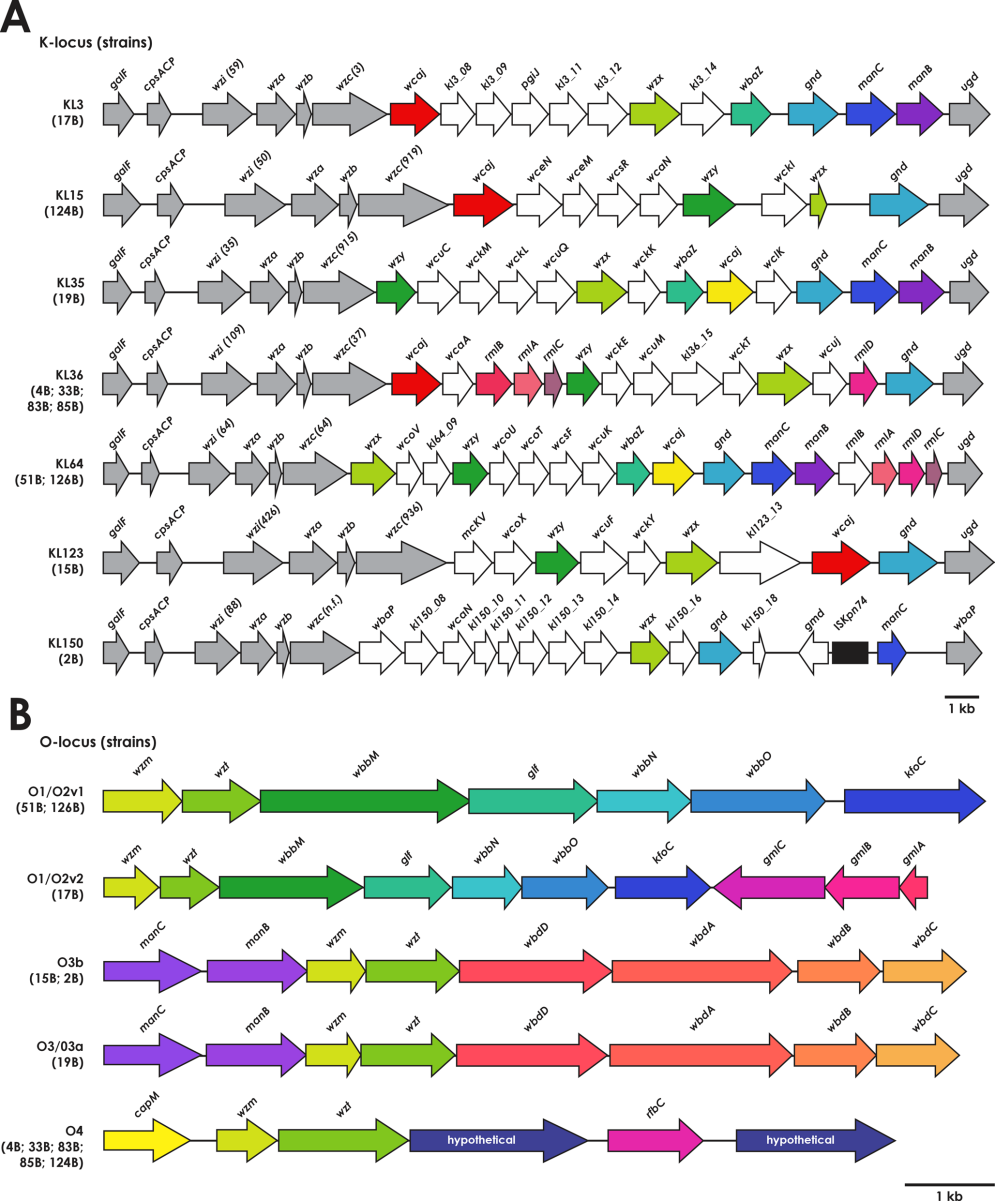
Representation of the structure and diversity of K-loci identified in CRKP strains. Coding sequences are represented by arrows. Gray arrows represent conserved genes at the 5’ and 3’ end of the K-locus. The *wzc* and *wzi* allele types are indicated above their respective arrows. The central region of the K-locus has a variable genetic constitution, with genes exclusive to each K-locus represented by white arrows, and genes present in multiple K-loci, with varying positions and sizes, represented by colored arrows. Same colors were maintained to represent the same gene across different K-loci. IS*Kpn74* present in strain 2B is represented by a black box

Seven K-loci were identified from the sequences of the capsular polysaccharide loci of the CRKP, namely: KL3, KL15, KL35, KL36, KL64, KL123, and KL150. KL36 was the most prevalent, being detected in strains from ST437 (strains 4B, 33B, 83B, and 85B). KL64 was identified in two strains from ST147 (51B and 126B), and the K-loci KL3, KL15, KL35, KL123, and KL150 were found in the strains 17B (ST394), 124B (ST340), 19B (ST384), 15B (ST628), and 2B (ST11), respectively (Fig. 1A).

The K-loci observed here exhibit a wide diversity of unique genes. Remarkably, the presence of the *gmd* gene, encoding a GDP-D-mannose 4,6-dehydratase, is exclusive to strain 2B (KL150) and located adjacent to the insertion sequence IS*Kpn74*, suggesting a potential rearrangement at the 3’ end of this K-locus (Fig. 1A).

Five distinct O-loci (Fig. 1B) were detected in the CRKP strains, namely: O1/O2v1, O1/O2v2, O3b, O3/O3a and O4. O4 was the most prevalent, being detected in the strains belonging to ST437 (4B, 33B, 83B, and 85B) and ST340 (124B). Next, O1/O2v1 was identified in strains from ST147 (51B and 126B), and O3b in strains from ST11 (2B) and ST628 (15B). The O-loci O1/O2v2 and O3/O3a were exclusive of strains from ST394 (17B) and 19B (ST384).

### Prevalence and Distribution of Virulence and Heavy Metal Resistance Genes in CRKP Strains

**Fig. 2:**
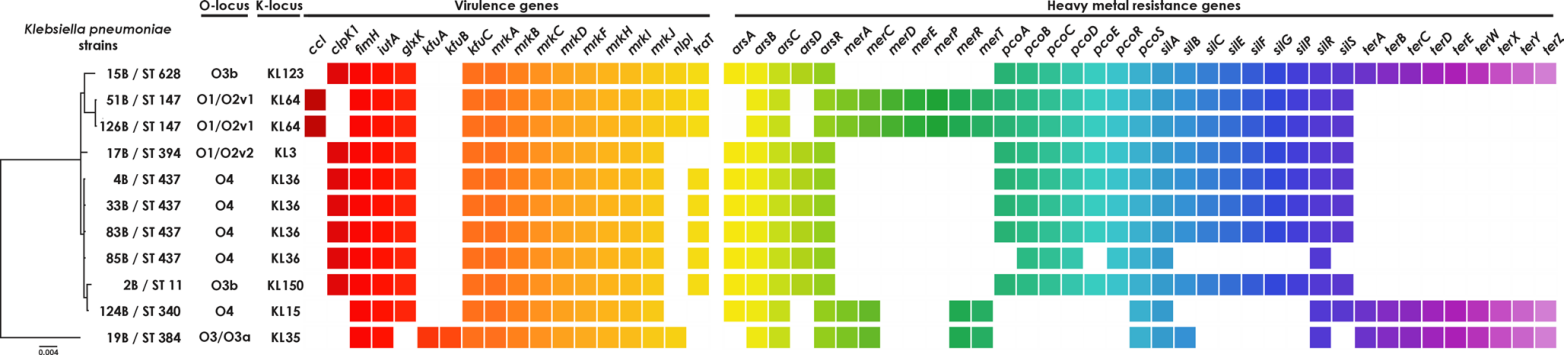
Virulome and heavy metal resistance cluster distribution in CRKP strains. Tree obtained by Bayesian Inference from the concatenated alignment of the seven housekeeping genes (*gapA*, *infB*, *mdh*, *pgi*, *phoE*, *rpoB*, *tonB*) used in multi-locus sequence typing (MLST). The identification of the K-locus and O-locus is indicated alongside the lineage identification and its respective ST. Genes conferring virulence (virulome) and heavy metal resistance are shown at top. A map according to the presence or absence of each virulence and heavy metal resistance gene is shown, where presence is indicated by colored boxes and absence by white

A total of nine groups of virulence features, comprising 18 genes, were detected among the CRKP strains, including (i) the bacteriocin colicin gene *ccI*, (ii) the heat shock survival AAA family ATPase gene *clpK1*, (iii) the type I fimbriae gene *fimH*, (iv) the ferric aerobactin receptor gene *iutA*, (v) the glycerate 3-kinase gene *glxK*, (vi) the ferric uptake system genes *kfuABC*, (vii) the mannose-resistant *Klebsiella*-like type III fimbriae cluster *mrkABCDFHIJ*, (viii) the lipoprotein NlpI precursor gene *nlpl*, and (ix) the outer membrane protein complement resistance gene *traT*. These genes were widespread among the strains, with an average number of 14 ± 0.55 virulence genes (Fig. 2).

The *ccI* gene was exclusive to the strains of ST147, specifically in strains 51B and 126B. Both strains exhibited a scaffold containing this gene with 100% coverage and 99.9% identity with the plasmid pIGMS32, a ColRNAI plasmid found in *K. pneumoniae*, which is 9294 bp in length (DQ298019.1).

The *clpK1* gene was observed in strains from ST11 (2B), ST394 (17B), ST437 (4B, 33B, 83B and 85B) and ST 628 (15B). Notably, in ST437 and ST394 strains, *clpK1* was associated with mobile genetic elements (MGEs), with ISK*pn*26 in ST437 and IS102 in ST394.

The *nlpl* gene was observed in strains from ST147 (51B and 126B), ST384 (19B) and ST628 (15B). The *fimH*, *iutA* and *kfuC* genes, and *mrkABCDFHIJ* cluster were detected in all studied CRKP strains. The plasmid-encoded *traT* gene was present in most strains, except for 17B (ST394), 19B (ST384) and 124B (ST340). Overall, the most frequent virulence factor profile was as follows: *clpK1*, *fimH*, *iutA*, *glxK*, *kfuC*, *mrkABCDFHIJ* and *traT* in CRKP strains from ST437 (4B, 33B, 83B and 85B) and ST11 (2B).

In strains from ST11 (2B), ST394 (17B), ST437 (4B, 33B, 83B, and 85B), and ST628 (15B), genes conferring resistance to arsenic, copper, and silver were carried by the same IncFII(K) plasmid. Notably, this plasmid also carried the virulence gene *clpK1*. The arsenic resistance cluster *arsABCDR* was complete in strains from ST628 (15B), ST394 (17B), ST437 (4B, 15B, 33B, 83B) and ST11 (2B), and incomplete in other strains. The copper resistance cluster *pcoABCDERS* and silver resistance cluster *silABCEFGPRS* showed similar distribution patterns, being complete in ST628 (15B), ST147 (51B and 126B), ST394 (17B), ST437 (4B, 33B, 83B), and ST11 (2B), and incomplete in other strains.

The mercury resistance cluster *merACDEPRT* was complete in strains from ST147 (51B and 126B), and incomplete (*merACRT*) in strains from ST340 (124) and ST184 (19B). The tellurium resistance cluster *terABCDEWXYZ* was complete only in ST340 (124B), ST384 (19B), and ST628 (15B) strains, carried by the IncHI1B plasmid (pNDM-MAR). Additionally, in the 19B and 124B strains, this plasmid also carried mercury resistance genes.

### Resistome of CRKP Strains

**Fig. 3:**
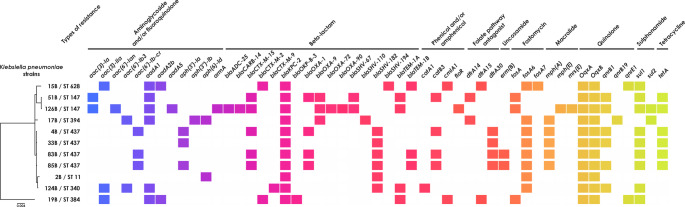
Distribution of the antimicrobial resistance genes in CRKP strains. Tree obtained by Bayesian Inference from the concatenated alignment of the seven housekeeping genes (*gapA*, *infB*, *mdh*, *pgi*, *phoE*, *rpoB*, *tonB*) used in multi-locus sequence typing (MLST). Genes conferring resistance (resistome) and their respective classes are shown at top. A map according to the presence or absence of each resistance gene is shown, where presence is indicated by colored boxes and absence by white

A total of 51 ARGs were identified in CRKP strains. On average, each strain harbored 14.7 ± 2.8 ARGs (Fig. 3). All strains possessed genes conferring resistance to three or more classes of antibiotics, which is consistent with their MDR phenotype.

Twelve genes associated with aminoglycoside and/or fluoroquinolone resistance were detected, the predominant genes being *aadA1* (54.5%), *aac(3)-IIa* (27.3%), *aac(6’)-Ib-cr* (27.3%), and *aph(3’)-Ia* (27.3%). Unique genes found in only one strain included *aac(6’)-Ian*, *aadA5*, and *armA* in strain 126B (ST147), and *aph(3’’)-Ib* in strain 17B (ST394).

Seventeen genes associated with β-lactam resistance were detected, including nine ESBL-associated genes conferring resistance to third-generation cephalosporins (*bla*_CTX−M−2_, *bla*_CTX−M−9_, *bla*_CTX−M−15_, *bla*_SHV−67_, *bla*_SHV−110_, *bla*_SHV−182_, *bla*_SHV−194_, *bla*_TEM−1 A_, and *bla*_TEM−1B_) and one gene conferring resistance to carbapenems (*bla*_KPC−2_). The *bla*_KPC−2_ gene was the most widespread, present in 100% of strains, followed by the ESBL-associated genes *bla*_SHV−182_ (54.5%), *bla*_CTX−M−15_ (45.5%), *bla*_TEM−1 A_ (36.4%) and the oxacillinase-encoding gene *bla*_OXA−1_ (36.4%). Unique genes found in specific strains: *bla*_ADC−25_, *bla*_CARB−14_, *bla*_OXA−72_ and *bla*_OXA−90_ in strain 126B (ST147), *bla*_OKP−B−3_ in strain 19B (ST384), and *bla*_CTX−M−9_ in strain 2B (ST11).

Chloramphenicol acetyltransferase *catA1* and *catB3* mediating phenicol resistance mediators were detected in 9.1% and 36.4% of the strains, and resistance to amphenicols was attributed to the presence of the *cmlA1* (19.2%) and *floR* (9.1%) genes. Dihydrofolate reductases associated with trimethoprim resistance were detected in 72.7% of the strains, with *dfrA30* being the most frequent gene (36.4%). Lincosamide resistance was observed in only 18.2% of the strains, characterized by the presence of the *erm(B)* gene. Fosfomycin resistance was ubiquitous, with all strains harboring at least one glutathione transferase gene in their genomes, with *fosA6* being the most frequent (72.8%).

Macrolide resistance genes were found in six strains (54.5%), with the *mph(A)* gene, encoding a 2’-phosphotransferase, being the most frequent (45.4%).

Quinolone resistance was universal, with all isolates harboring genes encoding the multidrug efflux RND (Resistance-Nodulation-Division) transporter periplasmic adaptor subunit *oqxA* and transporter permease subunit *oqxB* in their genomes. Plasmid-mediated quinolone resistance *qnr*-like determinants were detected in 81.8% of the strains, with *qnrB1* being the most frequent (54.5%).

Dihydropteroate synthases associated with sulfonamide resistance were detected in 90.9% of the strains, with *sul1* present in 81.8% and s*ul2* in only 18.2%. Only strain 126B harbored both *sul1* and *sul2* genes. Tetracycline resistance was due to the *tet(A)* gene among the ST147 and ST437 isolates, accounting for 54.5% of the isolates in this study.

All investigated CRKP strains showed a rich repertoire of regulators associated with the efflux pumps AcrAB, OqxAB, and AcrEF, a genotype also associated with resistance mechanisms in these pathogens. In addition, all strains exhibited point mutations in the *acrR*, *ompK36*, and *ompK37* genes, which may contribute to antimicrobial resistance through mechanisms such as efflux pump regulation (*acrR*) and reduced membrane permeability to β-lactams (*ompK36*), while the role of *ompK37* in carbapenem resistance remains unclear. Furthermore, except for strains 2B (ST11) and 19B (ST384), point mutations in the *ramR* gene were detected, without phenotypic conclusions. Mutations with inconclusive phenotypes were also identified in the *gyrB* gene in strains 19B (ST384) and 124B (ST340). The point mutations and regulatory variants identified in genes associated with antimicrobial resistance are summarized in Supplementary Table S1, including their distribution across the analyzed isolates.

### Genetic Context of *bla*_KPC−2_ in CRKP Strains

The genetic context of the *bla*_KPC−2_ gene is located on an IncN-type plasmid in strains of ST11 (2B), ST340 (124B), and ST437 (4B, 33B, 83B, 85B), and on an IncM1-type plasmid in strains of ST147 (51B and 126B), ST384 (19B), ST394 (17B), and ST628 (15B). Except for the ST11 (2B) strain, the *bla*_KPC−2_ gene is situated within a region that is 100% identical to the Tn*4401* transposon, with the following arrangement: *tnpR*-*tnpA*-IS*Kpn7*-*bla*_KPC−2_-IS*Kpn6*.

**Fig. 4:**
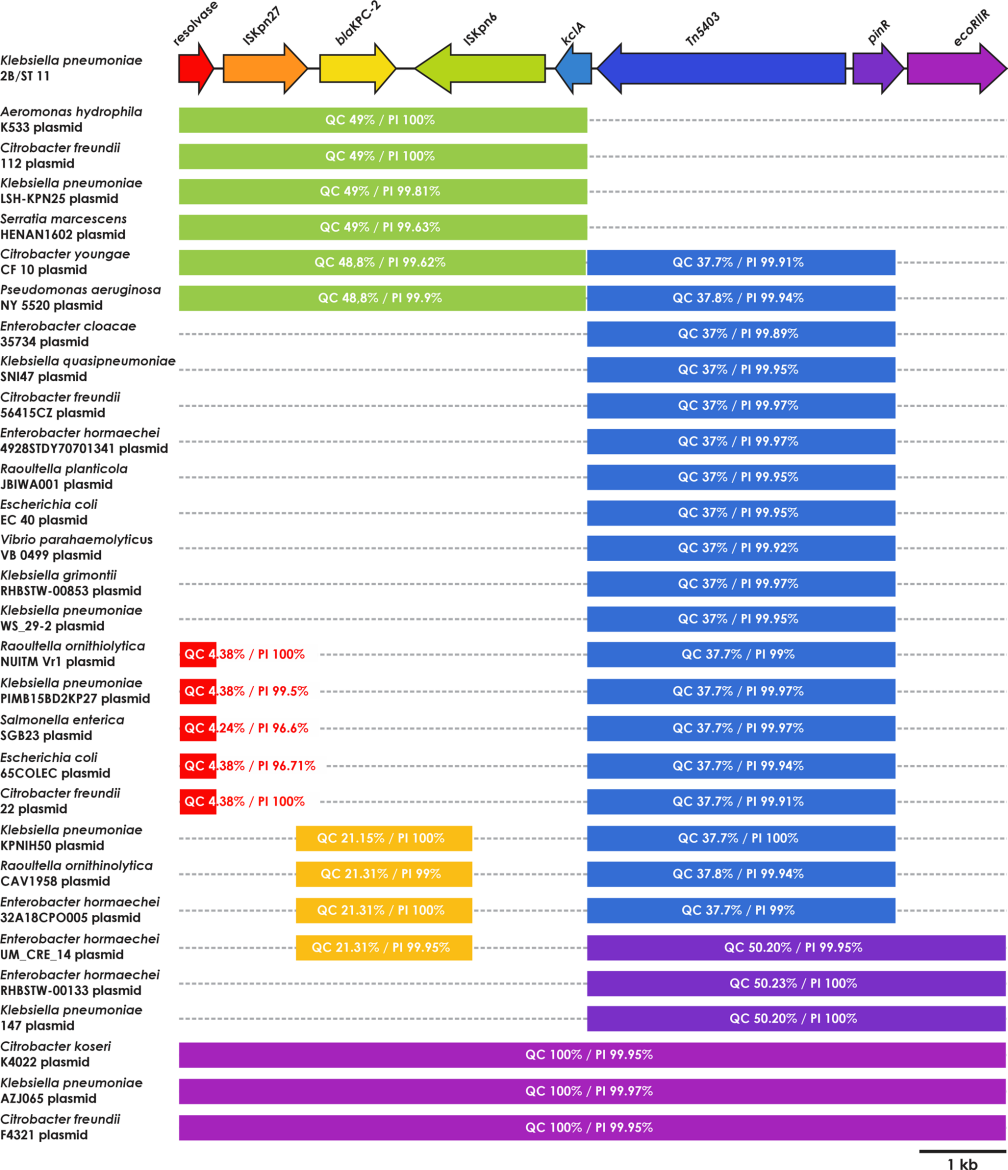
Representation and comparison of the unusual genetic context of the *bla*_KPC−2_ gene identified in this study. Coding sequences are represented by colored arrows. BLASTN analysis revealed regions of high sequence similarity between the replicon carrying the unusual *bla*KPC-2 context in the CRKP strain and plasmids from different Proteobacteria. This pattern is consistent with a putative recombination event contributing to the formation of this genetic structure. Query Cover (Q.C), Percent Identity (P.I)

The *bla*_KPC−2_ gene was found in an unusual genetic context for strain 2B (ST11), being as follows: resolvase-IS*Kpn27*-*bla*_*KPC−2*_-IS*Kpn6*-*KlcA*-Tn*5403*-*pinR*-*ecoRIIR* (Fig. 4). Comparison of this plasmid fragment with other sequences available at NCBI revealed extensive regions of similarity with different Proteobacteria plasmids. Furthermore, an alignment with 100% of coverage and 99.95% of identity was observed with *Klebsiella pneumoniae* plasmid AZJ065 (CP099521) as well as with *Citrobacter koseri* (CP136815), and *Citrobacter freundii* F4321 (CP137180), suggesting a putative recombination event between Proteobacteria plasmids, potentially resulting in a novel replicon carrying *bla*_KPC−2_ in *Enterobacterales* (Fig. 4).

## Discussion

The emergence of virulent CRKP strains in healthcare settings is a major challenge for global public health [21]. Therefore, knowing the genetic repertoire associated with virulence and antimicrobial resistance is of utmost importance for understanding the molecular evolution of these strains, as well as enabling the epidemiological surveillance of this pathogen. Here, we present significant findings on the virulome and resistome of CRKP strains exhibiting increased virulence prevalent in hospitals in southeastern Brazil. These isolates were previously classified as classical *K. pneumoniae*, as they lack key molecular markers commonly associated with hypervirulent strains, such as aerobactin and hypermucoviscosity regulators [8]. Although resistance genes were extensively characterized in this study, their presence does not necessarily translate into phenotypic resistance and should be interpreted with caution.

The genetic repertoire associated with *K. pneumoniae* virulence is vast and diverse, which makes it difficult to correlate it with in vivo virulence [8]. Although the strains analyzed in this study did not present genetic markers closely associated with the hypervirulence phenotype [22], it was observed that the 11 CRKP strains analyzed exhibit a dense virulome, similar to other clinical strains of this species [23, 24].

Capsule and lipopolysaccharide (LPS) are key virulence factors in *K. pneumoniae* [7]. The characteristics of K-loci and O-loci have proven important both for the epidemiological surveillance of *K. pneumoniae* and for providing information useful in the development of therapies to prevent and treat infections caused by this bacterium [14]. The CRKP strains analyzed here showed diverse K-loci; however, except for KL3 and KL64, these loci are uncommon [25].

The KL150 was identified in an ST11 strain in this study. This relatively rare K-locus was first described in a clinical isolate obtained in 2009 in Vietnam, also from ST11 with *wzi* 88 and KPC_− 2_ allele [26]. This K-locus harbors the *gmd* gene, involved in fucose biosynthesis and associated with *K. pneumoniae* virulence [27]. Here, *gmd* was identified in an inverted orientation adjacent to IS*Kpn*74, suggesting a rearrangement at the 3′ end of the locus.

Five O-loci were identified among the CRKP strains, with O4 being the most prevalent, particularly in ST437 and ST340 (GC258). Although O1 is typically the most common antigen in clinical isolates [14, 25], the predominance of O4 in this study aligns with its association with globally distributed MDR clones [14].

Other highly significant biomarkers with the potential to impact *K. pneumoniae* infections were observed in this study. Notably, all isolates harbored the *fimH* and *mrkABCDFHIJ* genes, which encode Type I and Type III fimbriae, respectively. These adhesins facilitate bacterial adhesion to epithelial and immune cells, as well as to abiotic surfaces [28]. Type I fimbriae are essential for initiating the adhesion process of *K. pneumoniae* to host tissues and are believed to contribute to virulence, while the type III fimbriae play a key role in biofilm formation, particularly in uropathogenic strains [29]. Furthermore, type III fimbriae are associated with outbreak-related *K. pneumoniae*, suggesting a role in persistence and transmission in clinical settings [24].

The ferric aerobactin receptor gene *iutA* and the *kfuC* component of the ferric iron uptake system, two key virulence factors, were detected in all isolates in this study. The *kfuABC* system, which is also prevalent in hypervirulent strains, has been associated with enhanced virulence by enabling bacterial lineages to access iron from a variety of human and environmental sources, thus potentially providing a competitive advantage in iron-limited environments encountered during infection [30].

The bacteriocin gene *cci*, which encodes colicin, was identified in two *K. pneumoniae* strains, both belonging to ST147. This gene was located on a plasmid, suggesting the potential for horizontal transfer within bacterial populations. *K. pneumoniae* produces various types of bacteriocins that have antimicrobial effects against closely related species; however, few studies have comprehensively reported the distribution of bacteriocins among the *Klebsiella* population [31]. While specific bacteriocins have been associated with certain STs of *K. pneumoniae*, this is the first report of a colicin-encoding gene in ST147, highlighting a unique adaptation within this lineage.

A high frequency of genes associated with heavy metal resistance was observed in CRKP strains, along with the co-occurrence of several of these gene clusters on the same plasmids, as reported in other studies [15, 32]. The co-occurrence of the mercury resistance cluster and the tellurium resistance cluster, was observed on the same plasmid, IncHI1B (pNDM-MAR). In addition, the co-occurrence of genes conferring resistance to arsenic, copper, and silver, along with the virulence gene *clpK1*, all located on the same IncFII(K) plasmid was observed. This co-occurrence not only facilitates the horizontal transfer of heavy metal resistance genes but also suggests that the selective pressure exerted by heavy metal exposure may favor the spread of virulence factors, increasing the pathogenic potential of these strains.

The identification of multiple ARGs in CRKP strains indicates a robust resistome and a significant genomic load of resistance determinants, enabling these pathogens to withstand multiple antibiotic classes. The presence of multiple ARGs within the same locus suggests that gene colocalisation may favor horizontal transfer, potentially accelerating the spread of resistance among different strains and species.

Here, all CRKP strains carried the *bla*_KPC−2_ gene, which encodes a carbapenemase responsible for high-level resistance to carbapenem antibiotics, including the *K. quasipneumoniae* strain. Carbapenem resistance can disseminate intraspecifically among the phylogroups of the *K. pneumoniae* complex. In this context, frequently misidentified species, such as *K. quasipneumoniae*, have been emerging as hosts of resistance genes, such as *bla*_KPC−2_ and *bla*_OKP−B−6_, and are associated with severe infections in hospital settings [33]. All strains exhibited *bla*_KPC−2_ in addition to ESBL genes, including members of the *bla*_CTX−M_; *bla*_OXA_; *bla*_TEM_; and *bla*_SHV_ variants. This same association, which has also been observed in CRKP strains in other studies, increases resistance to nearly all available antibiotics [34, 35].

The diversity of *bla*_KPC−2_ gene-carrying plasmids and their integration with other resistance genes was identified previously as responsible for the spread of MDR isolates [4]. The IncN plasmid was detected in CRKP strains belonging to ST11, ST340 and ST437. This finding highlights the broad dissemination capacity of this plasmid among CRKP strains, as previously demonstrated [24]. While in the ST147, ST384, ST394, and ST628 strains, the *bla*_KPC−2_ gene was identified in an IncM1-type plasmid. The IncM plasmid has frequently been misidentified and reported as belonging to the IncL/M group, which may account for the scarcity of reports regarding the presence of KPC-2 in *Enterobacterales* harboring IncM. The IncL/M group has primarily been associated with the *bla*_OXA−48_ gene [36]; however, in South America, it appears to facilitate the dissemination of the *bla*_KPC−2_ gene. Additionally, one study from China has also reported the IncL/M group as carrying this gene [37].

The *bla*_KPC_ gene is located on transferable plasmids and transposons, associated with insertion sequences (IS), thus facilitating the spread of inter and intraspecies resistance [38]. The result of this transposon-directed transfer can lead to different rearrangements and KPC plasmid types [39]. The most common mobile element containing *bla*_KPC_ is a transposon of the Tn*3* family, Tn*4401* [38]. Nine isoforms of Tn*4401* were identified, with Tn*4401*a and Tn*4401*b being the most frequent and best characterized [40, 41]. In this way, the association of *bla*_KPC_ variants with specific Tn*4401* isoforms can be used as a genetic marker to distinguish different plasmids carrying the *bla*_KPC_ gene [42].

In this study, it was observed that a region encoding *tnpR*-*tnpA*-IS*Kpn7*-*bla*_KPC−2_-IS*Kpn6*, corresponding to Tn*4401*, was conserved and found in most strains, suggesting that the *bla*_KPC−2_ gene could be transferred between different CRKP strains, not being restricted to a particular subtype or ST. Our results are consistent with previous studies in which the *bla*_KPC_ gene belonging to Tn*4401* was identified in isolates of different STs and *Enterobacteriaceae* species and *Pseudomonas aeruginosa* [43, 44]. Studies show that the presence of Tn*4401* in high-risk *K. pneumoniae* KPC-2-producing clones may play a key role in the spread of this gene among clinical pathogens [40, 45].

The 2B (ST11) strain showed the location of the *bla*_KPC−2_ gene in a different genetic context: resolvase-IS*Kpn27*-*bla*_*KPC−2*_-IS*Kpn6*-*KlcA*-Tn*5403*-*pinR*-*ecoRIIR*. The Tn*5403* transposon was initially identified as a “helper” element that collaborates with the transfer of non-conjugative plasmids found in *K. pneumoniae* isolates recovered from polluted aquatic environments [46]. Tn*5403* is known to contain only genes related to the function of transpositions and is known to transpose in a replicative fashion, appearing to act as a “reorganizing” force within plasmids of clinical isolates [47].

Recently, a study identified the spread of *bla*_KPC−2_ in different clones in strains of *K. pneumoniae* (not belonging to CC258), *K. variicola* and *K. quasipneumoniae* suggesting that the spread was related to the conjugative plasmid of the IncN type that carried the *bla*_KPC−2_ gene for Tn*4401*b modified through the insertion of a Tn*5403* element. Thus, it was speculated that this new structure could have originated through the introduction of the Tn*5403* element mediated by homologous recombination between hospital and environmental isolates and that this plasmid seems to have the ability to disseminate widely between hospitals [48]. This fact is worrying, since in our strains this transposon was found to belong to CC258, which is widely disseminated throughout the world.

The acquisition of the *bla*_KPC−2_ gene from *K. pneumoniae* ST11 followed a gradual evolutionary history rather than a one-step process, hypothesizing that the *K. pneumoniae* pandemic belonging to ST11 occurred because this ST had a better capacity of capturing or accumulating *bla*_KPC_ compared to the other types [49].

Therefore, it is suggested that the replicon containing an unusual *bla*_KPC−2_ context in a strain belonging to ST11 (CC258) may have originated from a recombination event between different Proteobacteria plasmids. These data reinforce the importance of using genomic tools for the epidemiological surveillance of CRKP, as these, in addition to contributing to the understanding of the molecular evolution of these strains, can help in tracking and controlling the spread of CRKP, which currently represents a major challenge for global public health.

## Conclusion

This study uncovers the complex genomic landscape of CRKP, showcasing a remarkable convergence of virulence and resistance traits mediated by mobile genetic elements. The diverse capsule and O-loci configurations, including rare variants associated with virulence, coupled with plasmid-encoded resistance mechanisms against antibiotics and heavy metals, underscore the adaptive versatility of these pathogens in clinical environments. Notably, the discovery of a novel insertion site carrying the *bla*_*KPC−2*_ gene in an ST11 (CC258) strain highlights the rapid molecular evolution within high-risk lineages. These findings underscore the pressing need for robust genomic surveillance to monitor and mitigate the spread of these highly adaptable pathogens.

## Supplementary Information

Below is the link to the electronic supplementary material.

Supplementary Material 1. Supplementary Table S1. Distribution of point mutations associated with antimicrobial resistance across the CRKP isolates analyzed in this study. The table presents the presence (1) or absence (0) of each mutation in individual strains, including nucleotide and amino acid changes, as well as their reported association with antimicrobial resistance when available.

## Data Availability

The BioProject accession number for the 11 MDR-CRKP strains is PRJNA1240238. The genome sequences of strains 2B, 4B, 15B, 17B, 19B, 33B, 51B, 83B, 85B, 124B, and 126B have been submitted to GenBank/NCBI under the following accession numbers: JBMHKA000000000, JBMHKB000000000, JBMHKC000000000, JBMHKD000000000, JBMHKE000000000, JBMHKF000000000, JBMHKG000000000, JBMHKH000000000, JBMHKI000000000, JBMHKJ000000000 and JBMHKK000000000, respectively.
